# Risk Factors for Cardiac Complications in Patients With Pheochromocytoma and Paraganglioma: A Retrospective Single-Center Study

**DOI:** 10.3389/fendo.2022.877341

**Published:** 2022-06-01

**Authors:** Lin Zhao, Xu Meng, QiMin Mei, Hua Fan, YeCheng Liu, XianLiang Zhou, HuaDong Zhu, ShuYang Zhang

**Affiliations:** ^1^Department of Cardiology, Fuwai Hospital, National Center for Cardiovascular Disease, Chinese Academy of Medical Sciences and Peking Union Medical College, Beijing, China; ^2^Department of Emergency, State Key Laboratory of Complex Severe and Rare Diseases, Peking Union Medical College Hospital, Chinese Academy of Medical Science and Peking Union Medical College, Beijing, China; ^3^Department of Urology, State Key Laboratory of Complex Severe and Rare Diseases, Peking Union Medical College Hospital, Chinese Academy of Medical Science and Peking Union Medical College, Beijing, China; ^4^Department of Cardiology, State Key Laboratory of Complex Severe and Rare Diseases, Peking Union Medical College Hospital, Chinese Academy of Medical Science and Peking Union Medical College, Beijing, China

**Keywords:** pheochromocytoma, paraganglioma, cardiovascular complications, hypertension, catecholamines (CAs)

## Abstract

**Background:**

Catecholamine excess arising from pheochromocytomas and paragangliomas (PPGLs) can cause a wide spectrum of cardiac manifestations. Although there are reviews of reported cases, these reviews lack detailed data, which makes it impossible to perform an accurate analysis. In this study, we conducted a comprehensive analysis of cardiovascular complications (CCs), including PPGL-related myocardial injury, cardiogenic shock, and arrhythmias requiring antiarrhythmic therapy, in a large cohort of patients with PPGL.

**Methods:**

We retrospectively analyzed the clinical data of consecutive patients with PPGL admitted between January 2018 and June 2020. The prevalence and the characteristics of patients with CCs were investigated. Moreover, comparisons were made between patients with and without CCs.

**Results:**

Compared with the non-CC group, the percentage of men was significantly lower (14/41 vs.92/175, 34.1% vs. 52.6%, p = 0.034) and the proportion of patients with paroxysmal hypertension was significantly higher (13/41 vs.29/173, 31.7% vs.16.8%, p = 0.03) in the CC group. More patients showed excessive sweating (19/41 vs 64/175, 46.3% vs. 24.0%, p = 0.004) and PPGL crisis (7/41 vs. 10/175, 17.1% vs.5.7%, p=0.035) in the CC group. In terms of laboratory findings, higher white blood cell [7.36 (6.49, 20.23) vs. 5.95 (5.1, 6.97)×10^9^/L, p<0.001] and platelet [339.28 ± 108.54 vs. 250.66 ± 70.83(×10^9^/L), p = 0.021] counts were more common in the CC group. There was also a higher prevalence of combination-producing PPGL in the CC group (13/24 vs.20/149, 54.2% vs.13.4%, p<0.001). However, the tumor size, invasive behavior on histology, and hemorrhage or necrosis on histology did not differ between the two groups. Platelet count [odds ratio (OR): 1.009; 95% confidence interval (CI) 1.001–1.016; p=0.023] and combination-secreting PPGL (OR: 5.009; 95% CI 1.365–18.38; p=0.015) are independent risk factors for CCs in patients with PPGL.

**Conclusions:**

In patients with PPGL, even in the absence of signs and symptoms of CCs, a work up of cardiology should be strongly considered. Importantly, if patients with PPGLs have higher platelet counts and the combination-secreting pattern, they are more likely to have CCs. Thus, a careful cardiac evaluation should be performed.

## Introduction

Pheochromocytomas and paragangliomas (PHEOs and PGLs, PPGLs) are a group of rare catecholamine (CA)-secreting neuroendocrine tumors that are separately derived from the adrenal medulla and the extra-adrenal sympathetic or parasympathetic nervous system (1). The joint annual incidence of PPGL is estimated to be 2–8 cases per million inhabitants (1). The clinical manifestations of PPGLs include hypertension (HTN), headache, excessive sweating, chest pain and so on, which are due to excessive CA secretion (2). However, the clinical presentation of PPGLs can also be asymptomatic and vague, which may explain the delay in diagnosis in many cases.

PPGLs are associated with a wide variety of cardiovascular complications (CCs), which have been reviewed previously (3). Briefly, CCs related to PPGLs include arrhythmias (3, 4),Takotsubo-like cardiomyopathy (5–7), dilated cardiomyopathy, and acute coronary syndrome and so on (3). Although thought to arise from the incident CA excess, the exact mechanism of CCs induced by PPGLs remains elusive (7). CCs in patients with PPGLs may be life-threatening; hence, timely diagnosis and surgery can be life-saving (3). Different types of CCs may occur in one fifth to more than one third of patients with PPGLs (4, 8–10). Given the potential reversibility of these CCs, early diagnosis and resection of PPGLs are crucial because delayed diagnosis may lead to irreversible cardiac remodeling and death. However, the diagnosis of PPGL-related CCs is often delayed due to the atypical presentation in many cases.

Although there are reviews of reported cases (11, 12), these reviews lack detailed data, which make it impossible to perform an accurate analysis, and publication bias due to the tendency to report more severe cases is inevitable (13, 14). In this study, we retrospectively reviewed patients diagnosed with PPGLs at a single center and screened these patients for CCs. The prevalence and features of PPGLs with CCs were assessed. Furthermore, comparisons between patients with and without CCs were made to identify the clinical features associated with CCs to aide in further understanding this clinical entity.

## Materials and Methods

### Study Population

Consecutive patients who were diagnosed with PPGLs and admitted to the hospital between January 2018 and June 2020 were included. PPGLs were diagnosed in 407 patients. Forty-six patients referred for recurrence or metastasis, 21 patients with pathologically confirmed non-PPGLs, 41 patients who did not undergo surgery because they had metastatic PPGL or refused surgery, 10 patients aged <18 years, 6 patients with congenital heart disease (1 with Kawasaki disease, 1 with left cor triatriatum, 1 with congenitally corrected transposition of the great arteries, 2 with atrial defects, and 1 with congenital single ventricle), 12 patients finally diagnosed with coronary heart disease, and 55 patients with incomplete clinical data were excluded. Thus, 216 patients with histopathological evidence were included in the analysis. The flowchart of patient recruitment is shown in **Supplementary Figure 1**.

### Clinical Assessment

Electronic health records were reviewed, and clinical history data, biochemical test results, imaging results, surgical reports, and pathology diagnoses were extracted and analyzed. Smoking history was defined as current smoking. Drinking history was defined as current drinking. Diabetes mellitus (DM) was defined as: 1) fasting plasma glucose value ≥ 7.0 mmol/L or a 2-h plasma glucose value ≥ 11.1 mmol/L during a 75 g OGTT or HbA1c ≥ 6.5% confirmed by repeated testing in an asymptomatic patient; or 2) random plasma glucose ≥ 11.1mmol/L in a patient with classic symptoms of hyperglycemia; or 3) preexisting diagnosis of DM with established antidiabetic diet/treatment (15). The patterns of HTN included normal, sustained (just as essential HTN), paroxysmal (paroxysms of HTN on a background of normal blood pressure [BP]), mixed (paroxysms of HTN on a background of sustained HTN), and unknown (16). HTN was based on the diagnosis at admission and preoperative BP readings, as BP can decline after surgery. A secreting tumor was defined by a CA or metanephrine concentration at least twice the upper limit of normal (ULN). The pattern of secretion was classified as non-secreting, epinephrine predominant (≥2 × ULN), norepinephrine predominant (≥2 × ULN), dopamine predominant (≥2 × ULN), or combination (epinephrine and norepinephrine ≥2 × ULN) (9). The maximum tumor diameters were comprehensively determined from gross pathological specimens. The estimated glomerular filtration rate (eGFR) was calculated using the CKD-EPI method (17).

CCs were diagnosed if at least one of the following clinical situations was present before surgery: (1) biochemical, electrocardiographic, and/or echocardiographic evidence of myocardial ischemia with left ventricular systolic dysfunction, with absence of significant coronary artery stenosis confirmed by coronary artery angiography or coronary computed tomography angiography (18); (2) heart failure of unknown etiology, but most likely attributed to CA release from PPGL, requiring hospitalization or intravenous diuretic therapy; (3) arrhythmia requiring hospitalization or antiarrhythmic therapy. The comparison group was composed of patients with PPGL who did not present with CCs.

All patient records were anonymized before analysis. The study protocol was approved by the ethics committee of the institution and followed the principles of the Helsinki Declaration.

### Statistical Analysis

Continuous variables were assessed for normality using the Shapiro–Wilk test and the Kolmogorov–Smirnov test. Continuous data are expressed as mean ± standard deviation or median (25th, 75th percentile), as appropriate. The mean values were compared using the two-tailed Student’s t-test or, in the case of non-normally distributed data, the rank-sum test. Categorical variables are presented as number (percentage), and differences were detected using Pearson’s chi-square test or Fisher’s exact test. The parameters identified as statistically significant in the univariate logistic regression analysis (p < 0.1) were included in the multivariate logistic regression analysis to identify independent predictors. Parameters with odds ratios (ORs) of >1 were considered as risk factors, whereas parameters with ORs of <1 were considered as protective factors. Two-sided p values of <0.05 were considered statistically significant. All analyses were performed using SPSS statistical software, version 25.0 (IBM Corp.).

## Results

### Characteristics of Patients With and Without CCs

The clinical profiles of patients with PPGL are summarized in **Table 1**. A total of 216 patients were included in the analysis. Among the study subjects, 41 patients with PPGLs had CCs, while 175 patients did not. Men and women with PPGL were roughly equally represented, while the percentage of men in the non-CC group (92/175, 52.6%) was significantly higher than in the CC group (14/41, 34.1%) (p = 0.034). There were no differences between the two groups in terms of age at diagnosis of PPGL, history of smoking and alcohol abuse. DM occurred in 48 patients (22.2%). As expected, more patients with CCs had DM (14/41 vs. 34/175, 34.1% vs. 19.5%, p = 0.043). Higher levels of plasma glucose [6.1 (4.8, 7.6) vs. 5.3(4.7, 6.1) (mmol/L), p = 0.025] were more common in the CC group.

**Table 1:** Clinical profiles of patients diagnosed with PPGL and comparisons between the non-CC and CC groups.

Indications	All (n=216)	Non-CC (n=175)	CC (n=41)	P value
**Age, years**	47 (32,54.25)	48 (32.25, 54.75)	44.5 (28.5, 54.75)	0.702
**Male, %**	106 (49.1)	92 (52.6)	14 (34.1)	0.034
**Body mass index, Kg/m^2^**	24.39 ± 3.66	24.56 ± 3.54	23.74 ± 4.14	0.094
**History of smoking, %(n=215)**	42 (19.5)	37 (21.3)	5 (12.2)	0.188
**History of drinking,%(n=214)**	22 (10.3)	19 (11.0)	3 (7.3)	0.683
**Diabetes, %**	48 (22.3)	34 (19.5)	14 (34.1)	0.043
**Concurrent tumor/cancer**	23 (10.6)	17 (9.7)	6 (14.6)	0.523
**Hypertension, %**	126 (58.9)	96 (55.5)	30 (73.2)	0.039
**Patterns of hypertension**				
**Sustained, %**	79 (36.9)	63 (36.4)	16 (39)	0.756
**Paroxysmal, %**	42 (19.6)	29 (16.8)	13 (31.7)	0.03
**Unknown, %**	5 (2.3)	4 (2.3)	1 (2.4)	1.000
**Symptoms**				
**Dizziness or headache, %**	85 (39.4)	65 (37.1)	20 (48.8)	0.17
**Palpitations, %**	83 (38.4)	64 (36.6)	19 (46.3)	0.247
**Excessive sweating, %**	61 (28.2)	42(24.0)	19 (46.3)	0.004
**Nausea or vomiting, %**	19 (8.8)	12 (6.9)	7 (17.1)	0.076
**Chest pain or chest tightness, %**	26 (12.0)	17 (9.7)	9 (22.0)	0.057
**Abdominal discomfort^1^, %**	20 (9.3)	13 (7.4)	7 (17.1)	0.106
**PPGL crisis, %**	17 (7.9)	10 (5.7)	7 (17.1)	0.035
**SBPmax, mmHg**	180 (160, 210)	180 (160, 200)	210 (160, 242)	0.111
**DBPmax, mmHg**	110 (100,128.5)	110 (100, 120)	129 (103.75,142.5)	0.025

Continuous variables are expressed as mean ± standard deviation or median (25th, 75th percentile); categorical variables are presented as number and percentages in parentheses. Missing data varied by variables. PPGLs, pheochromocytomas and paragangliomas; CCs, cardiovascular complications;SBPmax, the highest level of systolic blood pressure; DBPmax, the highest level of diastolic blood pressure; ACEI, angiotensin converting enzyme inhibitor; ARB, angiotensin receptor antagonist; CCB, calcium channel blocker.

^1^Abdominal discomfort mainly refers to abdominal pain, bloating, or other indescribable abdominal discomfort.

The results of BP measurements were obtained in 214 patients, including 41 in the CC group and 173 in the non-CC group. HTN occurred in 126 patients (58.3%), more patients with CCs had HTN (30/41 vs. 96/173, 73.2% vs. 55.5%, p = 0.039); the proportion of patients with paroxysmal HTN in the CC group was significantly higher than non-CC group (13/41 vs. 29/173, 31.7% vs 16.8%, p = 0.03). However, the sustained (p = 0.756) and unknown (p = 1.000) patterns of HTN did not differ between the two groups. The maximum systolic BP (SBP) and diastolic BP (DBP) values were higher in the CC group than in the non-CC group, but only the DBP was significantly different between the two groups (p = 0.025). We collected data on preoperative antihypertensive drug use in 211 patients, of whom 163 patients (77.3%) received alpha-adrenergic receptor blockers, including 28 in the CC group (28/37, 75.7%) and 135 in the non-CC group (135/174, 77.6%). Beta-receptor blockers were used in 67 patients (67/211, 31.8%), including 23 in the CC group (23/37, 62.2%) and 44 in the non-CC group (44/174, 25.3%). Calcium channel blockers were used in 9 patients (4.3%), including 1 in the CC group (1/37, 2.7%) and 8 in the non-CC group (8/174, 4.6%). Angiotensin converting enzyme inhibitors/angiotensin receptor antagonists were used in 3 patients (1.4%), all in the non-CC group. Patients took alpha-adrenergic receptor blockers for 7-14 days before surgery to normalize BP and heart rate. Preoperative coadministration of beta-receptor blockers was indicated to control tachycardia only after administration of alpha-adrenergic receptor blockers (19).

Regarding to the symptoms documented in medical records, 25 patients (25/216, 11.6%) were asymptomatic. Dizziness or headache, palpitations, profuse sweating, nausea or vomiting, chest pain or tightness, and abdominal discomfort appeared in 85 (39.4%), 83 (38.4%), 61 (28.2%), 19 (8.8%), 26 (12.0%), and 20 (9.3%) patients, respectively. PPGL crisis occurred in 17 patients (7.9%). Surprisingly, the proportions of patients who displayed dizziness or headache (65/175 vs.20/41, 37.1% vs. 48.8%, p = 0.17), palpitations (64/175 vs.19/41, 36.6% vs. 46.3%, p = 0.247), nausea or vomiting (12/175 vs.7/41, 6.9% vs. 17.1%, p = 0.076), chest pain or tightness (17/175 vs. 9/41, 9.7% vs. 22.0%, p = 0.057), or abdominal discomfort (13/175 vs. 7/41, 7.4% vs. 17.1%, p = 0.106) were similar between the two groups. Nevertheless, significantly more patients demonstrated profuse sweating (19/41 vs.42/175, 46.3% vs. 24.0%, p = 0.004) and PPGL crisis (7/41 vs. 10/175, 17.1% vs. 5.7%, p = 0.035) in the CC group. There were 3 patients presented with cardiogenic shock in the PPGL-CC group (3/41, 7.3%). PPGL was incidentally found in 72 patients (33.3%), and other symptoms (anxiety, sweaty palms, fever, etc.) were present in about 20% of our cohort.

In terms of laboratory findings, higher white blood cell (WBC) [7.36 (6.49, 20.23) vs. 5.95 (5.1, 6.97)×10^9^/L, p<0.001] and platelet (339.28 ± 108.54 vs. 250.66 ± 70.83(×10^9^/L), p = 0.021) counts were more frequently observed in the CC group (**Table 2**). Hemoglobin and eGFR were not significantly different between the two groups. The concentrations of 24-hour urine epinephrine, 24-hour urine norepinephrine and 24-hour urine dopamine were available for 173 patients, including 24 patients in the CC group and 149 patients in the non-CC group. Among them, the non-secreting pattern appeared in 64 patients (37.0%). The types of secretion were epinephrine predominant in 19 patients (11.0%), norepinephrine predominant in 56 patients (32.4%), and combination in 33 patients (19.1%). One case demonstrated a dopamine-predominant pattern. There was no difference in the frequency of non-secreting, epinephrine-predominant, or norepinephrine-predominant patterns between the two groups; however, a higher proportion of patients had combination-secreting pattern (13/24 vs 20/149, 54.2% vs. 13.4%, p <0.001) in the CC group (**Table 2**).

**Table 2:** Laboratory, electrocardiographic, and echocardiographic findings of patients diagnosed with PPGLs and comparisons between the non-CC and CC groups.

Indications	All (n=216)	Non-CC (n=175)	CC (n=41)	P value
**Blood parameters(n=216)**				
**White blood cell count, × 10^9^/L**	6.28 (5.21,7.41)	5.95 (5.1, 6.97)	7.36 (6.49,20.23)	<0.001
**Platelet count, × 10^9^/L**	269.21 ± 87.32	250.66 ± 70.83	339.28 ± 108.54	0.021
**Hemoglobin, g/L**	142 (128,152.5)	142 (126.5,149)	145 (134,169.5)	0.855
**Glucose, mmol/L**	5.3 (4.7,6.3)	5.3 (4.7, 6.1)	6.1 (4.8,7.6)	0.025
**eGFR, ml/min/1.73 m2**	100.55 (91.79, 111.83)	101.72 (93.64,113.15)	93.47 (55.94, 105.73)	0.218
**Patterns of secretion(n=173)**				
**Epinephrine predominant, %**	19 (11.0)	18 (12.1)	1 (4.2)	0.424
**Norepinephrine predominant, %**	56 (32.4)	46 (30.9)	10 (41.7)	0.294
**Dopamine predominant, %**	1 (0.6)	1 (0.7)	0 (0)	
**Combination, %**	33 (19.1)	20 (13.4)	13 (54.2)	<0.001
**ECG changes(n=211)**				
**ST-T segment changes, %**	32 (15.2)	6 (3.4)	26 (72.2)	<0.001
**QTc interval prolongation, %**	3 (1.4)	1 (0.6)	2 (5.6)	0.076
**Echocardiography (n=178)**				
**LVEF(%)**	68 (64, 72)	68 (65,72)	68 (60,72)	0.258
**Segmental ventricular wall motion abnormality, %**	3 (1.7)	0 (0)	3 (8.1)	
**Atrial or ventricular enlargement, %**	19 (10.7)	13 (9.2)	6 (16.2)	0.354
**Widening of ascending aorta, %**	18 (10.1)	17 (12.1)	1 (2.7)	0.170
**Decreased left ventricular diastolic function, %**	53 (29.8)	43 (30.5)	10 (27.0)	0.681
**Thickening of the ventricular septum, %**	3 (1.7)	2 (1.4)	1 (2.7)	0.505

Continuous variables are expressed as mean ± standard deviation or median (25th, 75th percentile); categorical variables are presented as number and percentages in parentheses. Missing data varied by variables.

PPGL, pheochromocytomas and paragangliomas; CCs, cardiovascular complications; ECG ,electrocardiogram; eGFR, estimated glomerular filtration rate.

Electrocardiography (ECG) was found in 211 of 216 patients (97.7%), including 175 patients in the non-CC group and 36 patients in the CC group. The most common ECG changes was ST-T change (15.2%) and it was more common in the CC group (6/175 vs. 26/36, 3.4% vs. 72.2%, p<0.001), while QTc prolongation (1/175 vs. 2/36, 0.6% vs. 5.6%, p = 0.076) presented similarly in patients with and without CCs. Arrhythmias occurred in 21 patients (21/211, 10.0%), including sinus tachycardia in 5 subjects, atrial fibrillation (AF) in 2 subjects, premature atrial beats in 8 cases, occasional premature ventricular beats in 3 patients, and atrioventricular block in 1 subject. Two patients had both premature atrial beats and premature ventricular beats. AF and atrioventricular block all occurred in the CC group. Atrial arrhythmias and ventricular arrhythmias were more likely in the CC group (CC group vs. non-CC group: 8/36 vs. 4/175, 22.2% vs. 2.3%, p<0.001; 4/36 vs.1/175, 11.1% vs. 0.6%, p=0.003, respectively). Among these, all occasional premature atrial beats occurred in the non-CC group, and three patients in CC group had frequent premature atrial beats. The incidence of sinus tachycardia was not statistically different between the two groups (4/175 vs. 1/36, 2.3% vs. 2.8%, p = 1.000). 178 patients in the study had echocardiograms. The two groups did not differ significantly in terms of the proportions of patients with atrial or ventricular enlargement (13/141 vs. 6/37, 9.2% vs. 16.2%, p = 0.354), widening of the ascending aorta (17/141 vs. 1/37, 12.1% vs. 2.7%, p = 0.170), decreased left ventricular diastolic function (43/141 vs. 10/37, 30.5% vs. 27.0%, p = 0.681), and thickening of the ventricular septum (2/141 vs. 1/37, 1.4% vs. 2.7%, p = 0.505) (**Table 2**).

Of the 216 patients, 104 patients (48.1%) had PGLs only, 111 patients (51.4%) had PHEOs only, and 1 patient had both (PPGL). Four patients (3.6%) had bilateral PHEOs. There was no statistical difference between PHEOs and PGLs for inducing CCs (p = 0.473). The proportion of bilateral PHEOs and the tumor size were not statistically different between the two groups. Regarding to the locations of PGLs, forty patients (40/216, 18.5%) were head and neck PGLs, including 8 (8/41, 19.5%) in the CC group and 32 (32/175, 18.3%) in the non-CC group; fifty-one patients(51/216, 23.6%) had intra-abdominal PGL, including 12 (12/41, 29.3%)in the CC group and 39 (39/175, 22.3%) in the non-CC group; nine patients (9/216, 4.2%) were bladder PGLs, including 2 patients (2/41, 4.9%)in the CC group and 7 patients(7/175, 4.0%) in the non-CC group; there were also 5 patients (5/216, 2.3%) with PGLs elsewhere, including 2 with cardiac PGLs, 1 with mediastinal PGL, 1 with bone PGL, and 1 with pelvic PGL. The locations of the PGL were not statistically different between the two groups. Invasive behavior and the presence of hemorrhage/necrosis on pathology were described in 6.0% and 9.7% of patients, respectively. More patients in the CC group demonstrated invasive behavior (4/41 vs. 9/175, 9.8% vs. 5.1%, p = 0.451) and hemorrhage/necrosis (7/41 vs.14/175, 17.1% vs. 8.0%, p = 0.141) on pathology than in the non-CC group, but they were not statistically significant (**Table 3**).

**Table 3:** Tumor characteristics of patients diagnosed with PPGLs and comparisons between the non-CC and CC groups.

Indications	All (n=216)	Non-CC (n=175)	CC (n=41)	P value
**Paragangliomas, %**	105 (48.6)	83 (47.4)	22 (53.7)	0.473
**Pheochromocytoma (n=112)**				
**Bilateral adrenal tumors, %**	4 (3.6)	4 (4.3)	0 (0)	1.000
**Unilateral adrenal tumors, %**	108 (96.4)	89 (95.7)	19 (100)	1.000
**Maximal tumor diameters (cm)**	5.5 (4.08, 7)	5.5 (4.13, 7)	5 (4.08, 6)	0.588
**Invasive behavior at histology, %(n=216)**	13 (6.0)	9 (5.1)	4 (9.8)	0.451
**Hemorrhage/necrosis at histology, %(n=216)**	21 (9.7)	14 (8.0)	7 (17.1)	0.141

Continuous variables are expressed as mean ± standard deviation or median (25th, 75th percentile); categorical variables are presented as number and percentages in parentheses. Missing data varied by variables.

PPGL, pheochromocytomas and paragangliomas; CCs, cardiovascular complications.

We also followed up 196 patients for 19.4 ± 11.3 months, and three patients were confirmed with metastatic PPGL (one each with pancreatic metastases, bone metastases, pulmonary and pelvic metastases). Twenty-three patients had concurrent tumors, including 17 in the non-CC group and 6 in the CC group, with no statistical difference between the two groups (17/175 vs. 6/41, 9.7% vs.14.6%, p=0.523). The types of concurrent tumors were medullary thyroid cancer (n=6, 26.1%), lung cancer (n=4, 17.4%), gastrointestinal stromal tumor (n=3, 13.0%), hematologic tumors (n=2, 8.7%), cervical cancers (n=2, 8.7%), ovarian borderline serous cystadenoma (n=1, 4.3%), ovarian teratoma (n=1, 4.3%), gastric cancer (n=1, 4.3%), kidney cancer (n=1, 4.3%), breast cancer (n=1, 4.3%) and meningioma (n=1, 4.3%).

### Potential Risk Factors for CCs in Patients With PPGL

To explore the potential risk factors for CCs among patients with PPGL, parameters with p values of <0.1 were included in the multivariate logistic regression analysis. The clinical symptoms of patients with PPGL occurred due to CA secretion. Thus, interactions among these parameters were possible. As such, only the patterns of secretion were included in the multivariate logistic regression analysis. Finally, sex, age, DM, BMI, maximum SBP, maximum DBP, WBC count, platelet count, hemoglobin, plasma glucose and secretion pattern (epinephrine, norepinephrine, and combination) were included in the multivariate logistic regression analysis. The multivariate logistic regression analysis showed that platelet count [OR: 1.009, 95% confidence interval (CI): 1.001–1.016, p = 0.023] and the combination pattern of secretion (OR: 5.009, 95% CI: 1.365–18.380, p = 0.015) were independent risk factors for CCs in patients with PPGL (**Table 4**).

**Table 4:** Multivariate logistic regression analysis of risk factors for CCs among patients with PPGL.

Variables	OR (95% CI)	P value
**Platelet count**	1.009 (1.001–1.016)	0.023
**Combination-secreting pattern**	5.009 (1.365–18.38)	0.015

OR, odds ratio; 95%CI, 95% confidence interval; PPGL, pheochromocytomas and paragangliomas; CCs, cardiovascular complications.

## Discussion

In this retrospective study, we summarized our experience of PPGL associated with CCs. Our study provides insights into the frequency, clinical characteristics, and predictors of PPGL associated with CCs. We observed a relatively high prevalence (almost 19.0%) of CCs in patients with PPGL, suggesting the universality of CA-related cardiac damage. Some abnormalities that did not require medical treatment on ECG, including sinus tachycardia, premature complexes, and non-specific ST-T changes, were not classified as CCs because they are non-specific for PPGL-related cardiac damage; hence, the occurrence rate of CCs might have been underestimated. To our knowledge, we are the first to identify that platelet count and the combination-secreting pattern are independent risk factors for CCs in patients with PPGL.

In a recent study, the rate of classical triad symptoms in patients with PPGL was as low as 17% and less than 10% of PPGLs in general has no symptoms at all (8). In our study, 11.6% of the patients had no clinical symptoms, this result was consistent with what has been reported (8, 20). About 20% of the patients had mild nonspecific symptoms, such as anxiety, sweaty palms, etc., this should remind clinicians not to rely on the classical triad as a diagnostic threshold. Three patients (1.4%) presented with cardiogenic shock in our study, which was similar to the reported rates (5, 21). Unfortunately, we could not conduct further statistical analyses due to the small number of cases. However, there was an interesting phenomenon that Sattler et al (22)found a trend towards a lower risk for cardiogenic shock in Takotsubo cardiomyopathy (TTC) patients taking beta-blockers at admission. This may suggest that beta-blocker intake prior to the TTS event might protect against the deleterious effects of CAs (23). However, further studies are needed to determine the role of beta-blockers in this situation.

Due to the rarity of PPGL, many patients were only screened in the presence of characteristic symptoms. However, because of the widespread use of cross-sectional imaging, the main method used to identify PPGL has changed; in this study, 33.3% of PPGL cases were identified incidentally. However, in recent studies, there are about 60% of PPGLs were discovered incidentally (8, 24). The reason for the low proportion of incidental PPGLs in our data may be due to the fact that computed tomography (CT) is not carried out as a routine physical examination in China and patients may refuse to undergo CT due to concerns about the damage of radiation to the body; the recent emphasis on the treatment of HTN, has increased attention to the reasons of secondary HTN, wider knowledge of PPGLs means that doctors in our hospital are more likely to consider PPGLs as a differential diagnosis in patients with non-specific symptoms, which helps to improve the diagnosis of PPGLs.

In our study, 22.2% of the participants had DM, which corresponds to previously published data (15, 25, 26). Some researchers have suggested the pathogenesis of DM in PPGL is multifactorial and includes impaired insulin secretion and insulin deficiency, defects in insulin signalling and response, as well as increase endogenous glucose production and impaired exogenous glucose uptake (27). Novel contributing mechanisms are also emerging, such as the indirect effects of CAs on insulin sensitivity through adiponectin (27).DM is known to be a risk factor for cardiovascular diseases (28), which may explain the more patients in PPGL-CCs group suffered from DM. Sattler. et al (23) presented a strong association between TTC and malignant diseases, which was due to the high catecholaminergic state. Screening for concurrent tumors is therefore recommended for all patients with PPGL. Notably, PPGL can be one clinical phenotype of specific clinical syndromes, such as multiple endocrine neoplasia type 2(MEN2) (29). This may lead to unique features in the distribution of comorbid tumor types in PPGL. For example, in our study, medullary thyroid carcinoma was the most common concurrent tumor in PPGL patients who were subsequently diagnosed with MEN2. Therefore, we recommend timely concurrent tumors screening for PPGL patients, especially for tumors related to PPGL-related syndromes.

The total proportion of arrhythmias in PPGL patients in this study was similar to Zelinka ‘s study (4). There were only 2 patients suffering from AF. However, previous studies (30, 31) have shown that the in-hospital morbidity and mortality rates were significantly higher in TTC with than without AF (31). TTC and PPGL-TTC are believed to have similar physiopathology (5, 6, 32), and excessive CAs have also been reported to play an important role in the occurrence of adverse arrhythmias (33–36).Therefore, it is important to pay attention to the arrhythmias in PPGL. Although QTc interval prolongation in PPGL is rare, it has been reported in several cases (37, 38). The QTc prolongation could be also explained by elevated catecholamines, which can increase late sodium current and reduce the transient outward current (33, 34).

Notably, norepinephrine-predominant secretion was present in most patients with PPGL, and combination secretion was present in most patients with CCs. PPGLs with epinephrine-predominant secretion may present with hypotension and shock due to interplay between multiple factors, including intravascular volume depletion, abrupt cessation of CA secretion due to tumor necrosis, desensitization of adrenergic receptors, and hypocalcemia (21, 39). In the current study, almost half of the patients had PHEOs, with a similar proportion of PHEOs to PGLs as the reported studies (9, 40). However, another study showed that PGLs were less common than PHEOs (41). This discrepancy may be due to the limitations of retrospective single-center studies. For example, the locations of the PGLs, such as retroperitoneal or mediastinal, may make surgery difficult, and patients were thus often referred to our hospital for surgery, which may have increased the relative ratio of PGLs. Besides, since head and neck PGLs were included in our study, this did increase the proportion of PGLs. In agreement with the recognition that all PPGLs have a metastatic risk, we reported a higher rate of invasive behavior on pathology in PPGLs with CCs (9.8%), but the results of our study did not reach statistical significance. This may be due to the lack of standardized interpretation of the pathology results. Also, the presence of hemorrhage/necrosis on histology was more often seen in subjects with CCs than in those without CCs, this result is consistent with previous studies (10, 41). This conclusion should thus be cautiously promoted, and more research is necessary for definitive clarification.

It has been frequently maintained that PPGL-associated CCs are rare (42, 43). In the present study, almost 19.0% of patients with PPGL presented with CCs. Recent studies have also shown that the probability of CCs in PPGL is 10.8%-20.9% (10, 41, 44), and our results are consistent with this. To explore the potential risk factors for CCs among patients with PPGL, we conducted the multivariate logistic regression analysis. We found that a higher platelet count and a combination-secreting pattern were independent risk factors for CCs in patients with PPGL. Platelets play an interesting role in the development of CCs in PPGL. Human platelets express both adrenergic and dopaminergic receptors (45, 46). CAs modulate thrombopoiesis (47) and platelet function (48) through platelet α2-adrenergic or dopaminergic receptors. High concentrations of CAs are sufficient alone to induce human platelet aggregation, granule secretion, and release of platelet markers (49). In addition, high levels of epinephrine also increase the number of circulating platelet, which has been attributed to an adrenaline action on spleen blood flow (46). Platelet activation and aggregation are critically involved in the pathophysiology of various diseases such as HTN, DM and atherosclerosis (50, 51). Clinical data also support the role of platelets in regulation of the immune response. The inappropriate and activation of a proinflammatory reaction has been linked to atherosclerosis (52). These theories may explain that increased platelet count in PPGLs is a risk factor for CCs in patients with PPGLs.

The pathogenesis of CCs associated with PPGL is generally thought to be similar to that of CA cardiomyopathy (7, 53). The pathophysiology of CA excess in the myocardium includes functional hypoxia due to increased contractility and coronary spasm leading to decreased blood flow, increased oxygen consumption due to excessive free fatty acid-induced mitochondrial uncoupling, intracellular calcium excess, stimulation of cell growth and cardiomyocyte hypertrophy, induction of interstitial fibrosis and scarring, chronic inflammation, direct toxicity, and generation of oxidative stress (54–56). However, CAs are unlikely to be the only mediators of CCs because some patients with very high levels of biochemical markers did not have CCs; in addition, individual patients respond variably to CAs, which may be due to the differential expression of adrenergic receptors (57, 58). The real etiologic link of PPGL-CCs remains unclear and seems to be multifactorial.

Our study has several limitations. First, the sample size was small. However, this is related to the rare incidence of PPGL. Second, bias was inevitable based on the retrospective and single-center study design. For example, BP classification was based on 24-hour ambulatory BP measurements if available, or otherwise on medical records. If paroxysmal BP elevation was detected during the course of the disease by the patients or by the medical staff during hospitalization, the patient was then classified into the “paroxysmal HTN” group. The limitations of retrospective studies mean that this classification may under-record patients with paroxysmal HTN. Third, cardiac magnetic resonance imaging (MRI) was not systematically evaluated. Relying solely on echocardiography may have resulted in some cases of myocardial involvement being missed. However, the limited availability of cardiac MRI makes it unsuitable for use as primary imaging modality for all patients. Fourth, the possibility of reporting bias with overestimation of uncharacteristic presentations and non-representative cases cannot be ruled out. This is again unavoidable due to the infrequent occurrence of this disease. Finally, these results should be generalized with caution owing to center-specific referral bias.

## Conclusion

PPGL can lead to life-threatening events and sometimes death; however, timely diagnosis and surgery can be life-saving. In all patients with PPGLs, even in the absence of signs and symptoms of CCs, a work up of cardiology should be strongly considered. Importantly, if patients with PPGLs have higher platelet counts and the combination-secreting pattern, they are more likely to have CCs. Thus, a careful cardiac evaluation should be performed.

## Data Availability Statement

The raw data supporting the conclusions of this article will be made available by the authors, without undue reservation.

## Ethics Statement

The studies involving human participants were reviewed and approved by the Ethics Committee of Peking Union Medical College Hospital. The requirement for informed consent was waived because of the retrospective nature of the study.

## Author Contributions

ZL, MX, and LYC designed the study. ZL collected patients'data. ZL, MX, and MQM performed the analyses and wrote the paper. FH assisted with data collection and analysis. ZXL, ZHD, and ZSY assisted with the study design. All authors contributed to manuscript revision, read, and approved the submitted version.

## Funding

This work was supported by the Science and Technology Innovation 2030 “New Generation Artificial Intelligence” Major Project (Ministry of Science and Technology) (2020AAA0109600), the Non-profit Central Research Institute Fund of Chinese Academy of Medical Sciences (2019XK320057), the CAMS Innovation Fund for Medical Sciences (2016-I2M-1-002), and the National Key Research and Development Program of China (2016YFC1300100). The funding sources had no involvement in the study design; the collection, analysis, and interpretation of data; the writing of the report; or the decision to submit the article for publication.

## Conflict of Interest

The authors declare that the research was conducted in the absence of any commercial or financial relationships that could be construed as a potential conflict of interest.

## Publisher’s Note

All claims expressed in this article are solely those of the authors and do not necessarily represent those of their affiliated organizations, or those of the publisher, the editors and the reviewers. Any product that may be evaluated in this article, or claim that may be made by its manufacturer, is not guaranteed or endorsed by the publisher.
