# Supplementary material for: Risk Factors for Cardiac Complications in Patients With Pheochromocytoma and Paraganglioma: A Retrospective Single-Center Study
Source: Front Endocrinol (Lausanne). 2022 Jun 1;13:877341. doi: 10.3389/fendo.2022.877341 (PMC9199364; doi:10.3389/fendo.2022.877341)
Supplement: Supplementary file 1 [file Image_1.pdf]

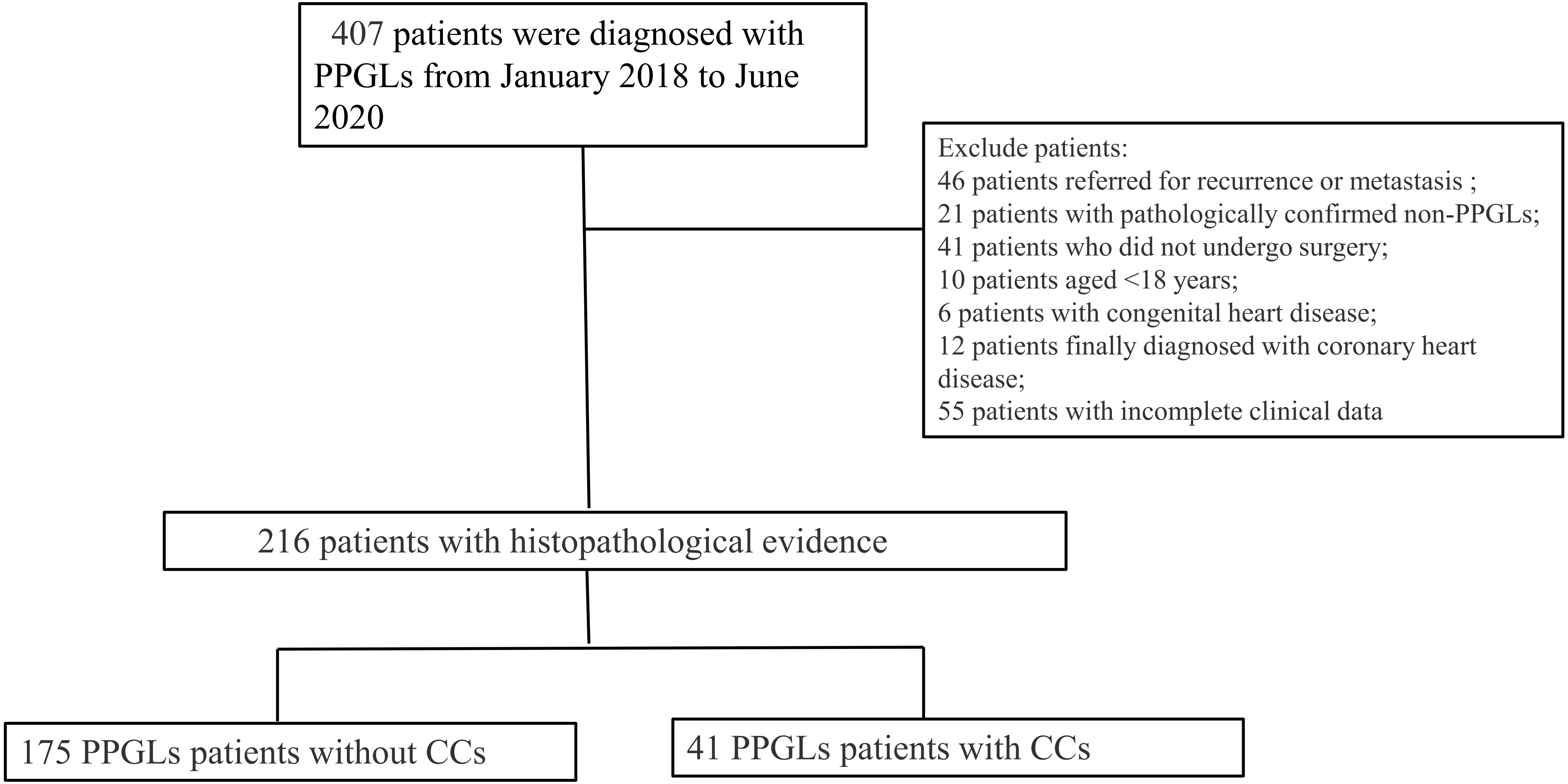

Supplementary Figure 1.Flow chart depicting the selection of patients with PPGL with and without CCs.
PPGLs: pheochromocytomas and paragangliomas; CCs: cardiac complications.
